# Zombi: a phylogenetic simulator of trees, genomes and sequences that accounts for dead linages

**DOI:** 10.1093/bioinformatics/btz710

**Published:** 2019-09-30

**Authors:** Adrián A Davín, Théo Tricou, Eric Tannier, Damien M de Vienne, Gergely J Szöllősi

**Affiliations:** 1 MTA-ELTE Lendület Evolutionary Genomics Research Group, Budapest, Hungary; 2 Department of Biological Physics, Eötvös Loránd, Budapest, Hungary; 3 Univ Lyon, Université Lyon 1, CNRS, Laboratoire de Biométrie et Biologie Évolutive UMR5558, Villeurbanne F-69622, France; 4 INRIA Grenoble Rhône-Alpes, Montbonnot-Saint-Martin F-38334, France; 5 Evolutionary Systems Research Group, Centre for Ecological Research, Hungarian Academy of Sciences, Tihany H-8237, Hungary

## Abstract

**Summary:**

Here we present Zombi, a tool to simulate the evolution of species, genomes and sequences in silico, that considers for the first time the evolution of genomes in extinct lineages. It also incorporates various features that have not to date been combined in a single simulator, such as the possibility of generating species trees with a pre-defined variation of speciation and extinction rates through time, simulating explicitly intergenic sequences of variable length and outputting gene tree—species tree reconciliations.

**Availability and implementation:**

Source code and manual are freely available in https://github.com/AADavin/ZOMBI/.

**Supplementary information:**

Supplementary data are available at *Bioinformatics* online.

## 1 Introduction

Reconstructing the pattern of horizontal gene transfers between species can help us date the origin of different taxa (Davín *et al.*, 2018; Wolfe and Fournier, 2018), understand the spread of genes of clinical importance (Lerminiaux and Cameron, 2019) and resolve difficult phylogenetic questions, such as inferring the rooting point of prokaryotic trees (Abby *et al.*, 2012; Szöllősi *et al.*, 2012; Williams *et al.*, 2017) or the evolutionary position of certain lineages of unclear origin (Boussau *et al.*, 2008). In the last decades, a large number of simulators have been developed to model a wide range of evolutionary scenarios (Beiko and Charlebois, 2007; Carvajal-Rodríguez, 2008; Dalquen *et al.*, 2012; Kundu and Bansal, 2019; Mallo *et al.*, 2016; Sjöstrand *et al.*, 2013) but none so far have considered the existence of extinct lineages and the horizontal transmission of genes (by lateral gene transfers) involving species that are not represented in the phylogeny (Fournier *et al.*, 2009; Szöllősi *et al.*, 2013; Zhaxybayeva and Peter Gogarten, 2004). Zombi simulates explicitly the genome evolution taking place in these extinct lineages, which is expected to have an impact in extant lineages by means of Lateral Gene Transfers (Szöllősi *et al.*, 2013). By not considering extinct lineages, other simulators make the implicit assumption that the transfer donor always leaves a surviving descendant among sampled species, while we know that this is most often not true (Szöllősi *et al.*, 2013). Making this assumption may potentially hamper our ability to simulate realistic scenarios of evolution. In addition to considering evolution along extinct lineages, Zombi includes several features hitherto not found together in any other simulator (Supplementary Table S1).

## 2 Basic features of Zombi

**Fig. 1.**
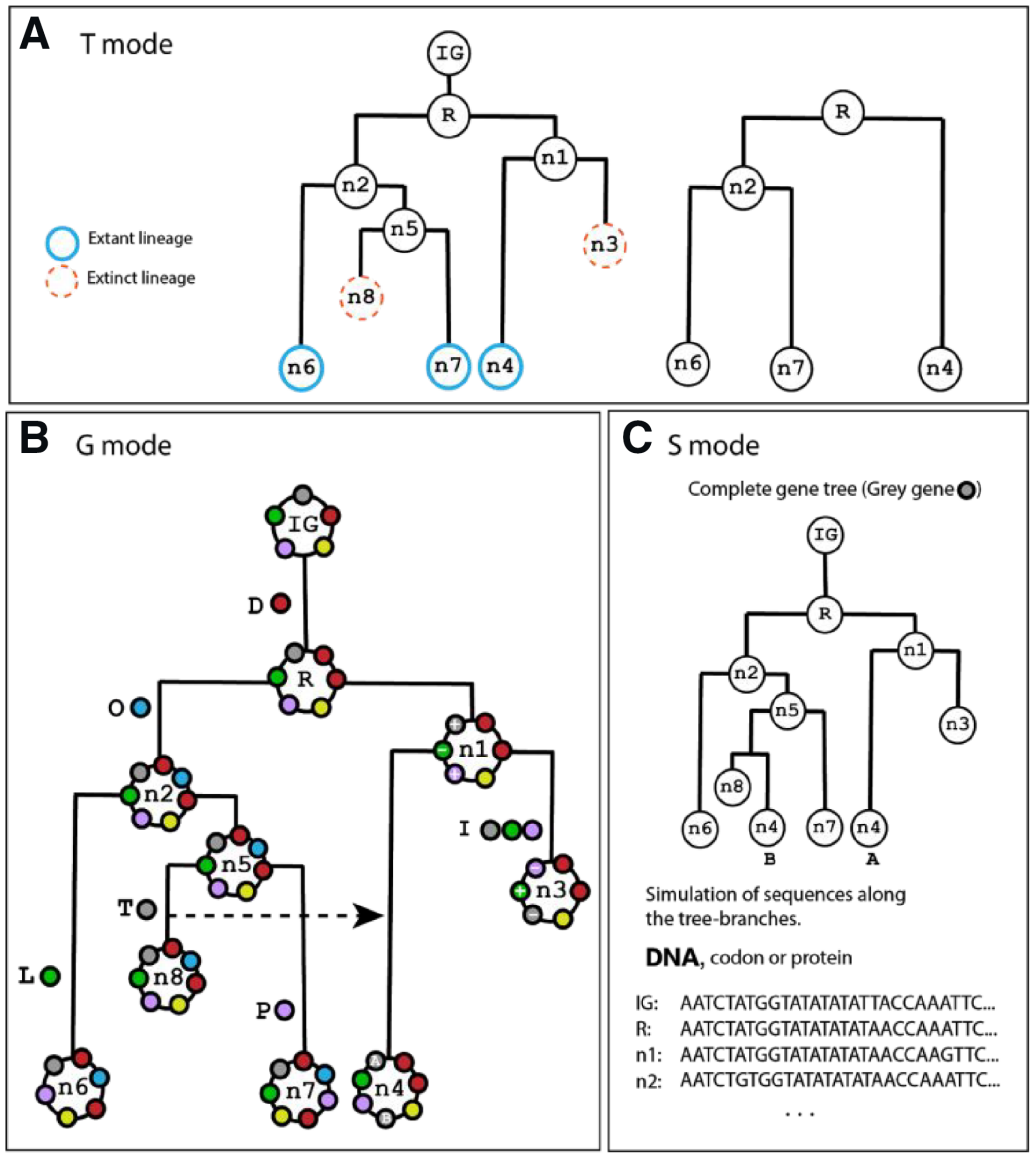
Overview of the three steps of the Zombi simulator. (**A**) In T mode, Zombi simulates a species tree using a birth-death process and outputs the pruned version of it by removing extinct lineages. In this example, lineages n3 and n8 go extinct before the simulation ends. (**B**) in **G** mode, a circular genome evolves within the branches of the complete species tree obtained with the **T** mode by Duplications (D), Originations (O), Inversions (I), Transpositions (P), Losses (L) and Transfers (T) of genes. The simulation starts with the initial genome (IG) containing a number of genes determined by the user (5 in this example, represented by the coloured circles). Each gene has an orientation (+ or -) that is determined randomly and represents the direction of the gene in the coding strand. Several events affecting different genes and their impact on the genome structure are indicated next to the branches where they occur. The inversion events not only modify the positions of the genes but also change their orientation. (**C**) In **S** mode, Zombi can be used to simulate codon, nucleotides and amino acids along the branches of the gene family trees. Here, the gene tree of the grey coloured gene family from B has been depicted

Zombi is a multilevel simulator, where a species tree is first simulated, then genomes evolve along the branches of this species tree, and finally, sequences are generated for each genome. These three steps, depicted in Figure 1 and detailed hereafter, are controlled by three main ‘modes’, named T, G and S, for species Tree, Genome and Sequence, respectively.

The T mode simulates a species tree under the birth-death model (Kendall, 1948), using the Gillespie algorithm (Gillespie, 1977), which is the standard method for simulating arbitrarily complex continuous time Markov processes (Supplementary Fig. S1). While more efficient and accurate methods exist to simulate the reconstructed tree (Hartmann *et al.*, 2010), taking into consideration unrepresented (extinct and unsampled) species requires simulating the complete species tree, which includes all extinct and unsampled branches of the phylogeny (Szöllősi *et al.*, 2013). This tree is subsequently pruned to obtain the reconstructed tree, by removing all the lineages that did not survive until the end of the simulation (Fig. 1A).

The **G** mode simulates the evolution of genomes within the branches of the complete species tree (Fig. 1B) using also the Gillespie algorithm (Supplementary Fig. S2) to account for six possible genome-level events: duplications, losses, inversions, transpositions, transfers and originations. Each of the first five events is characterized by two parameters: the first one is the effective rate, that controls the frequency and fixation probability; the second one controls the extension, i.e. the number of contiguous genes simultaneously affected by the event. Originations of new genes occurs one by one and therefore only a single effective rate parameter is needed. When a Transfer event occurs, the recipient lineage is randomly chosen from all the lineages alive at that time. The user can make the frequence of transfers to be higher between closely related lineages (Ochman *et al.*, 2000) (Supplementary Fig. S3). Once the simulation reaches the end, Zombi outputs a list containing each event that has occurred in the simulation for every gene family (all genes that share a common origin). Besides, the gene trees of each family are reconstructed by combining both species-level events (Speciations and Extinctions) and genome-level events (Duplications, Transfers and Losses). Inversions and transpositions do not modify the topology of the tree but add an extra layer of complexity by changing the neighborhood of genes, which is especially relevant when genome-level events affect more than one gene at a time (Supplementary Fig. S4). The gene family trees are also pruned to present the user the trees that can be expected to be recovered from most real-data analyses, removing all extinct lineages and gene branches that do not arrive until the present time. The **S** mode, finally, simulates gene sequences (at either the codon, nucleotide or protein level) along the gene family trees (Fig. 1C). The user can modify the scaling of the tree to better control the number of substitutions that take place per unit of time, and thus simulate fast or slow-evolving genes.

## 3 Advanced features

In addition to the basic features presented above, ‘advanced’ modes of Zombi (listed in Supplementary Table S2) can be used to obtain richer and more realistic evolutionary scenarios. For example, it is possible to use a species tree input by the user, to generate species trees with variable extinction and speciation rates, or to control the number of living lineages at each unit of time (Supplementary Fig. S5). At the genome level, Zombi can simulate genomes using branch-specific rates (Gu mode, allowing the user to simulate very specific scenarios such as one in which a certain lineage experiences a massive loss of genes), gene-family specific rates (Gm mode, which makes easier the process of using rates estimated from real datasets) and genomes accounting for intergenic regions (Gf mode) of variable length [drawn from a flat Dirichlet distribution (Biller *et al.*, 2016)]. At the sequence level the user can fine-tune the substitution rates to make them branch specific. Zombi provides the user with a clear and detailed output of the complete evolutionary process simulated, including the reconciled gene trees with the species tree in the RecPhyloXML reconciliation standard (Duchemin *et al.*, 2018).

## 4 Performance and validation

Simulations with Zombi are fast: with a starting genome of 500 genes and a species tree of 2000 taxa (extinct + extant), it takes around 1 min on a 3.4Ghz laptop to simulate all the genomes (Supplementary Fig. S6).

We validated that the distribution of waiting times between successive events was following an exponential distribution (Supplementary Figs S7 and S8), that the distribution of intergene sizes at equilibrium was following a flat Dirichlet distribution, as expected from Biller *et al.* (2016) (Supplementary Fig. S9), that the number of events and their extension occur with a frequency according to their respective rates (Supplementary Fig. S10) and that the gene family size distribution followed a power-law when duplication rates are higher than loss rates and stretched-exponential in the opposite case (Reed and Hughes, 2003; Szöllosi and Daubin, 2011) (Supplementary Fig. S11). We also checked by hand the validity of many simple scenarios to detect possible inconsistencies in the algorithm.

## 5 Implementation

Zombi is implemented in Python 3.6. It relies on the ETE 3 toolkit (Huerta-Cepas *et al.*, 2016) and the Pyvolve package (Spielman and Wilke, 2015). It is freely available at https://github.com/AADavin/ZOMBI along with detailed documentation and two tutorials in a wiki page.

## Supplementary Material

btz710_Supplementary_Data
