# Supplementary material for: Zombi: a phylogenetic simulator of trees, genomes and sequences that accounts for dead linages
Source: Bioinformatics. 2019 Sep 30;36(4):1286–8. doi: 10.1093/bioinformatics/btz710 (PMC7031779; doi:10.1093/bioinformatics/btz710)
Supplement: btz710_Supplementary_Data [file btz710_supplementary_data.pdf]

Name	Species Tree Level	Genome Level	Sequence Level	Extinct lineages	Sampling	Intergenic regions	Reconciled trees	Gene fusion-fission	ILS
Zombi	●	●	●	●	●	●	●		
ALF	●	●	●					●	
SimPhy	●		●				●		●
EvolSimulator	●		●						
GenPhyloData	●		●						
SaGePhy	●		●					●	

Table S1: Comparison of the features available in the main evolution simulators. Zombi (this paper), ALF (Dalquen et al. 2011), SimPhy (Mallo, De Oliveira Martins, and Posada 2016), EvolSimulator (Beiko and Charlebois 2007), GenPhyloData (Sjöstrand et al. 2013) and SaGePhy (Kundu and Bansal 2019). The features presented are whether the tool is capable of simulating species trees (Species Tree level), genomes (Genome level, meaning that it considers the structure of the genome, i.e. the physical adjacencies of genes in a genome), sequences (Sequences level), the presence of extinct lineages (Extinct lineages), the possibility of sampling species integrated in the simulator and pruning gene trees according to the species sampled (Sampling), the simulation of intergenic regions (Intergenic regions), outputting reconciled trees (Reconciled trees), considering fusion and fission of genes (Fusion-fission of genes) and producing ILS-induced gene tree/species tree discrepancy (ILS).

Mode	Description
**Species Tree**	
T	Basic mode
Tb	Branch-wise extinction/speciation rates
Tp	Lineage profiling (controls the number of extant lineages per unit of time)
Ti	Input tree by the user
**Genomes**	
G	Basic mode
Gu	Branch-wise event rates defined by the user
Gf	Simulate full genomes, including intergenic regions
Gm	Family-wise event rates defined by the user
**Sequences**	
S	Basic mode
Su	Branch-wise substitution rates defined by the user
Sf	Simulate sequences in combination with the Gu mode

Table S2. Zombi modes. Zombi implements a total of 11 different modes assigned to three main categories (Species Tree, Genome and Sequence). The basic mode of each category is explained in the main text of this paper.

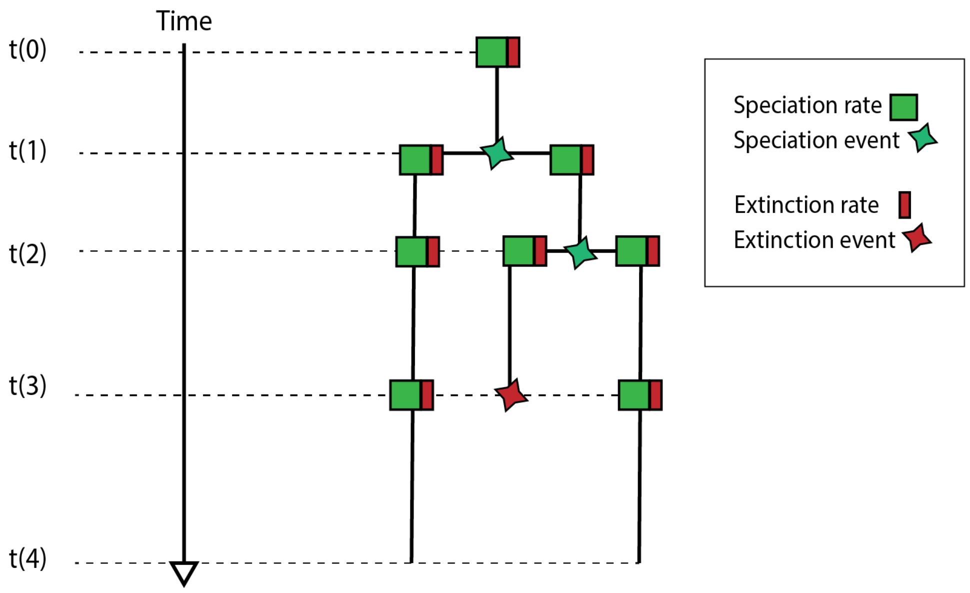

Figure S1. The Gillespie algorithm at the Species Tree level. At these level, there are two possible events: Speciations and Extinctions. Zombi starts with a single lineage at t = 0. To compute the time of occurrence of the next event, a number (t') is sampled from an exponential distribution with a rate equals to the sum of the rates of the individual event times the number of active branches. Then, a branch is chosen at random from all active branches, and the specific event that it undergoes is chosen according to their relative weights. All the active lineages increase their branch length in t' units and a new t' is computed repeating the same procedure. The number of active lineages increases by 1 in the case of a Speciation event or decreases by one in the case of an Extinction. The simulation stops until the total number of lineages reaches a number chosen by the user or the total length of the tree from the initial position to the active leaves attains a certain distance, also controlled by the user. To avoid dead lineages and speciation at the very end of the simulation, the last step of the simulation only increases the branch- length of all the active lineages but does not introduce a new event.

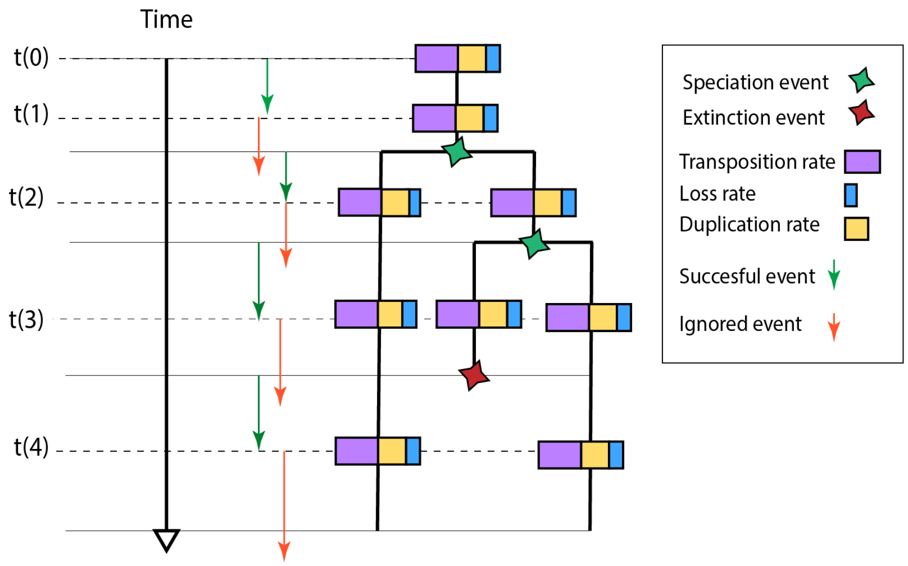

Figure S2. The Gillespie algorithm at the Genome level. A different set of events is used to model the Genome evolution (Duplications, Transfers, Losses, Transpositions, Inversion and Originations). In this example, rates of Origination, Inversion and Transfers are set to 0. The simulation starts at t(0), when the occurrence of the next event is determined by sampling from an exponential distribution with a parameter equal to the sum of all the rates of the active lineages (represented by the squared colours). The underlying patter of speciations and extinctions of the Species Tree is taken into account. If the number sampled form the exponential distribution is smaller than the time remaining for the next Species Tree level event, the event is considered successful and the genome affected is chosen randomly from all active lineages. Then, the specific event taking place is determined according to its rate, as well as its extension, and the affected genes are chosen randomly from all possible contigous positions in the genome. If the event is not successful, it is simply ignored. When a Speciation occurs (determined by the structure of the Species Tree), two identical genomes are created and they continue to evolve independently along the descending branch. Extinction event inactivates the genome evolving within that branch.

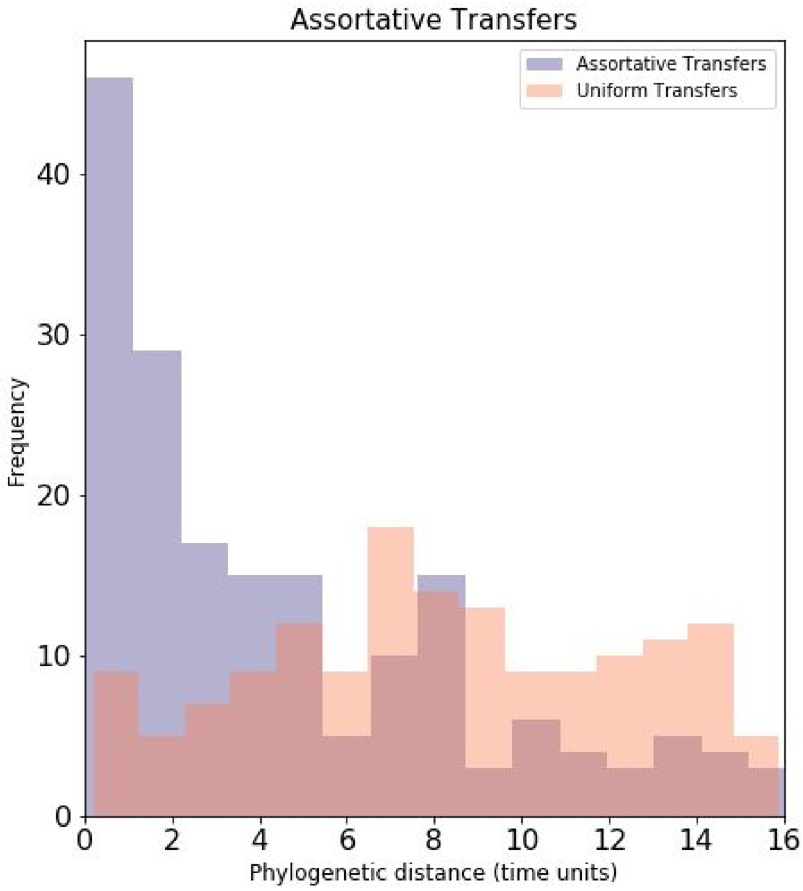

Figure S3. Assortative transfers. By default, when a transfer event occurs, it takes place between two randomly sampled lineages. The user can activate the function assortative transfer, which makes the transfer between two lineages to occur with a probability = $e^{-\alpha\delta}$ (being α a parameter to control for the strength of the effect and δ the normalized phylogenetic distance). The δ between two nodes is defined as the distance (in time units) between each of the nodes to their common ancestor. In this example, we simulate two datasets in the same Species Tree (30 species). The parameter α was set to 100. We can see how the assortative model of transfer makes transfers between closely related lineages more frequent than the uniform model, a phenomenon that has been observed in real data (Ochman et al. 2000).

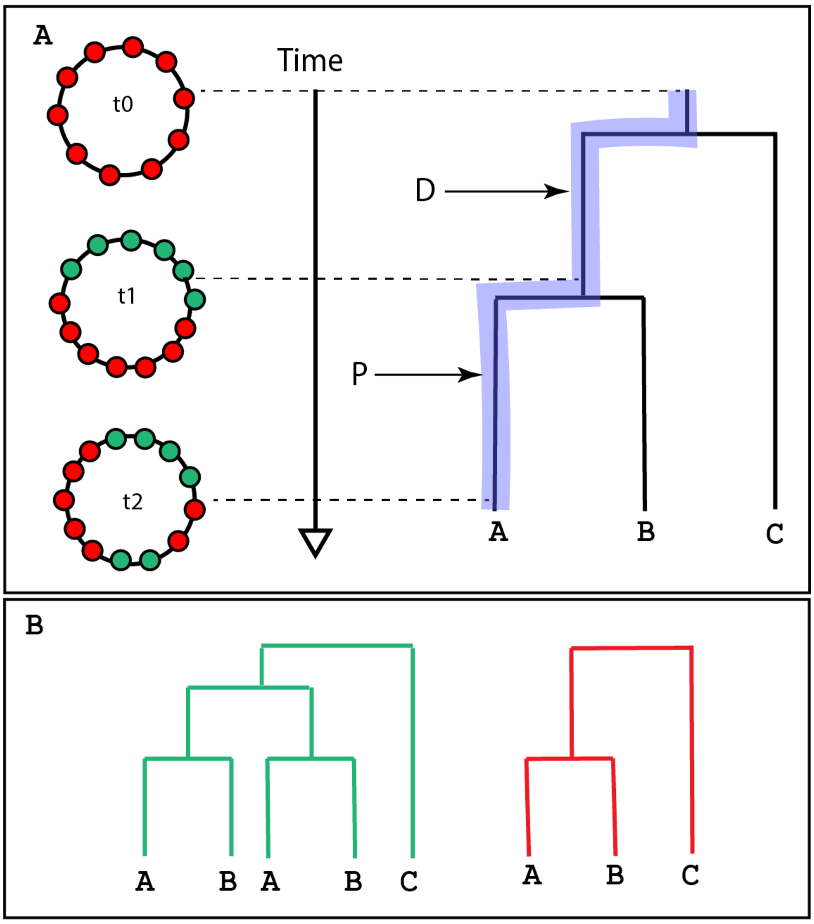

Figure S4. Gene position in Zombi. Simulators like SimPhy (Mallo, De Oliveira Martins, and Posada 2016) consider that all gene families evolve independently one from another. In **Zombi** a single event can affect more than one gene simultaneously. In A we have three snapshots of the genome evolving in the Species Tree on the right, along the blue branches. At time 0, none of the genes has undergone any event. At time 1, the green genes have undergone a duplication. At time 2, some red genes have been transposed within the duplicated genes. In B, the resulting gene trees from this scenario. We can see that in the resulting genomes the genes that, due to the transposition event, share the same duplication event, are shuffled with those that do not. Although inversions and transposition do not alter the topology of the gene trees directly, they can have a big impact when the different events affect more than one gene at a time.

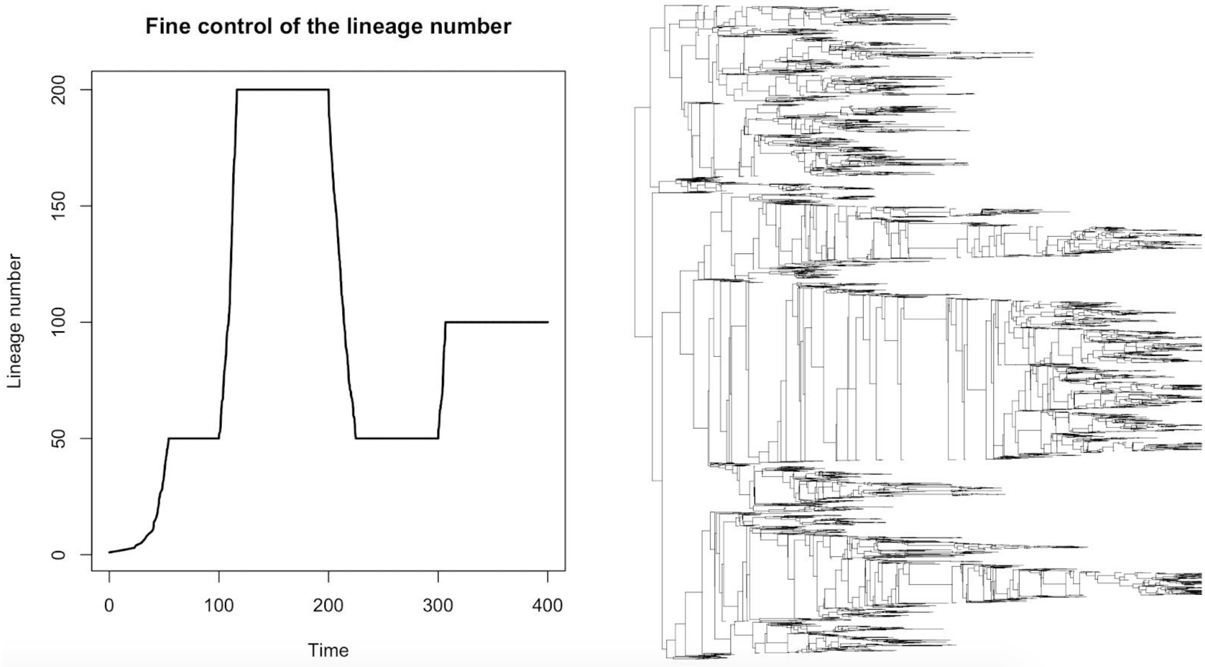

Figure S5. Fine control of the lineage number. Zombi can compute species tree using as input a list of times and the corresponding lineage number that should be attained by that time (in the example t 100 = 50; t 200 = 200; t 300 = 50; t 400 = 100). Zombi tries to attain the lineage number specified for each time interval using the speciation and extinction rates input by the user. At first, there is 1 living lineage and only speciations take place until the number of lineages = 50, number attained in this example when t~50. After that, and because time < 100, the number of lineages reaches an equilibrium in which there is a turnover of species controlled by a parameter also input by the user. Each time that a turnover event takes place two species are randomly sampled in the phylogeny. The first species undergoes a speciation and the second one dies, thus maintaining the total lineage number. The simulation continues until time = 400. In the right panel we can find the resulting species tree.

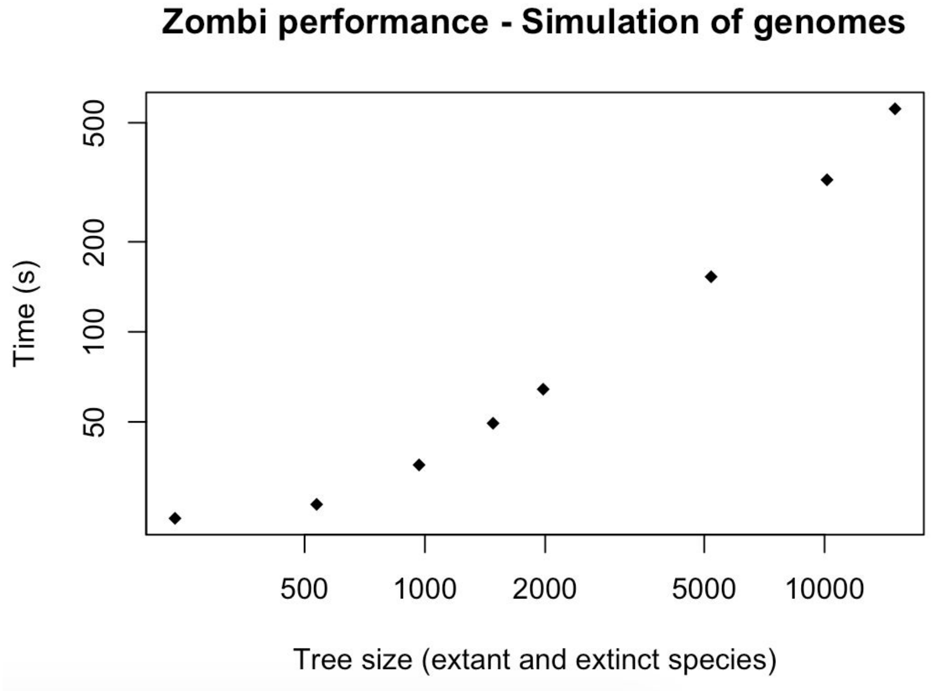

Figure S6. Computing time for different simulation in a computer with a 3,4 GHz Intel Core i5 processor. The rates used were Duplication rate: 0.2, Transfer rate: 0.2, Loss rate: 0.6, Origination rate:0.05, Inversion rate: 0.2, Translocation rate: 0.2. The initial genome was composed of 500 genes. All extension rates were set to 1. Species trees were obtained using by setting Speciation rate: 1 and Extinction rate: 0.5.

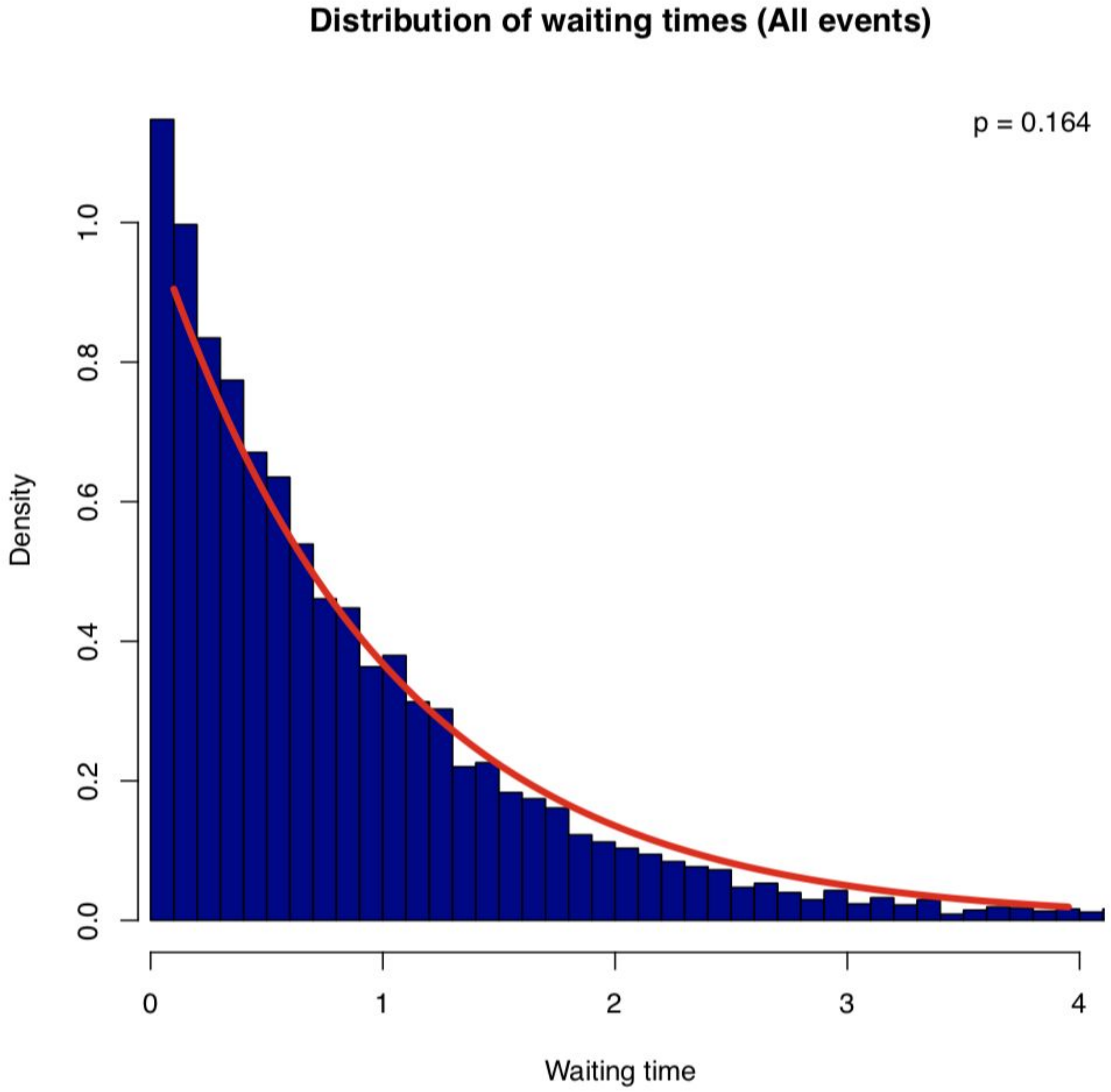

Figure S7. Comparison between the observed distribution of waiting times between consecutive events (duplications, transfers, losses, inversions, translocations and originations, blue bars) and the expected one (red line). The p-value corresponds to a KS test between the empirical distribution and an exponential distribution of the same rate than the same one used in the simulations.

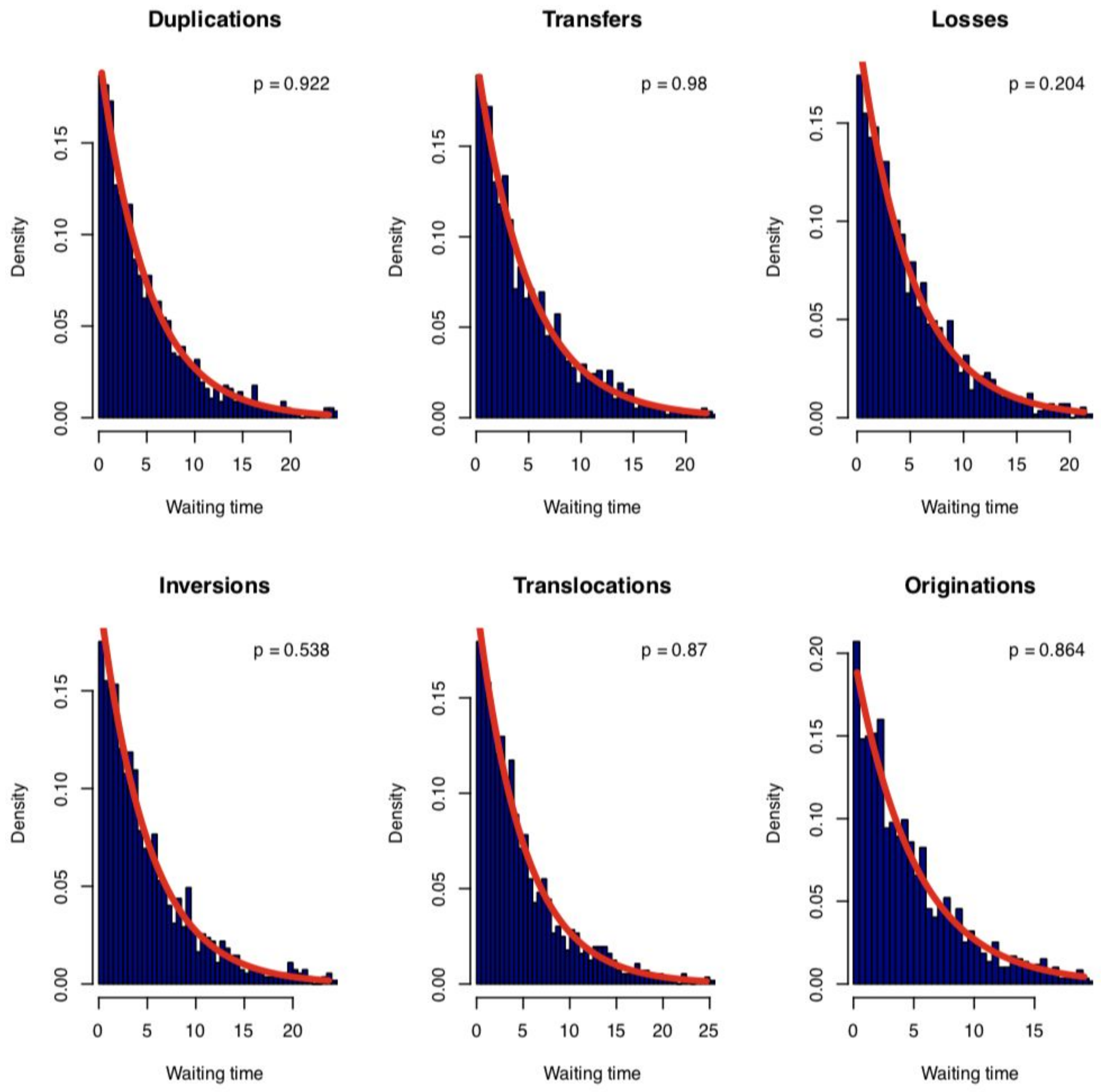

Figure S8. Comparison between the observed (blue bars) and the expected (red line) distribution of waiting times between consecutive events of each type. The p-value corresponds to a KS test between the empirical distribution and an exponential distribution of the same rate.

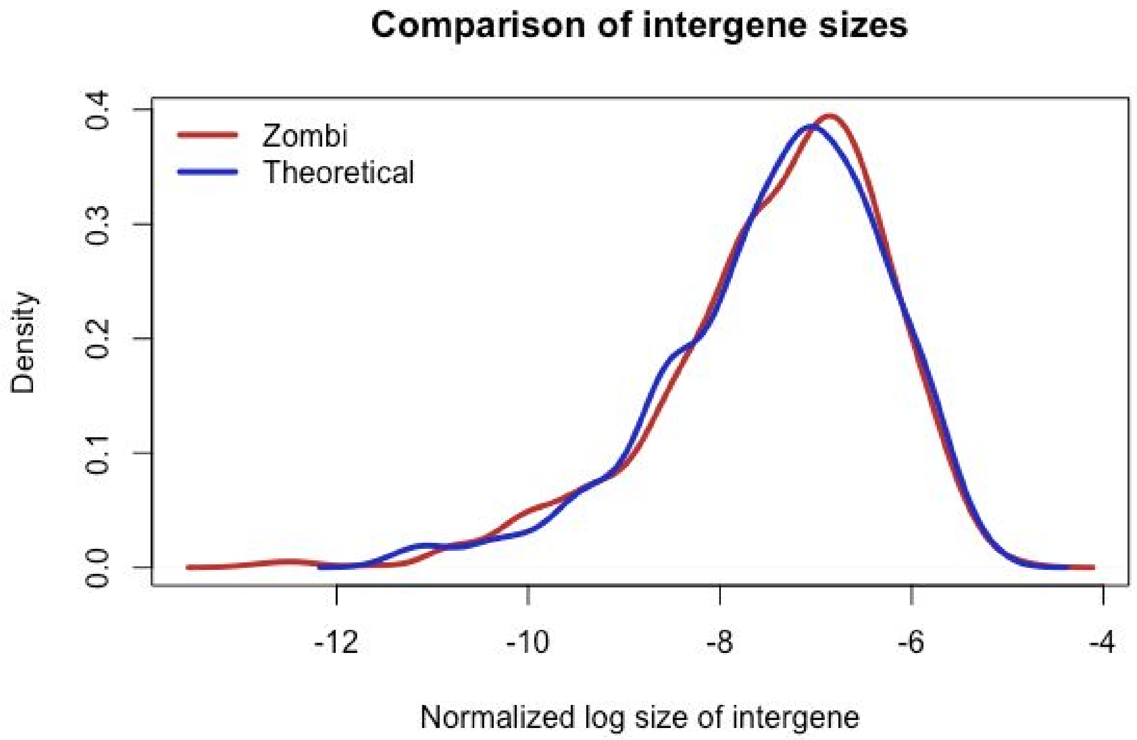

Figure S9: Validation of the mode Gf. To validate the mode **Gf** we simulated a genome with 1000 genes, whose intergene lengths had a constant size of 10000 nucleotides (instead of the Dirichlet as the default option). Then, we made it evolve under many inversion events (~10^6), to see whether at the equilibrium the intergene sizes followed a flat Dirichlet distribution, as expected (see Biller et al. 2016). We compared the obtained values with a randomly generated flat Dirichlet distribution using a KS test and obtained no significant difference (K-S test; p-value = 0.876)

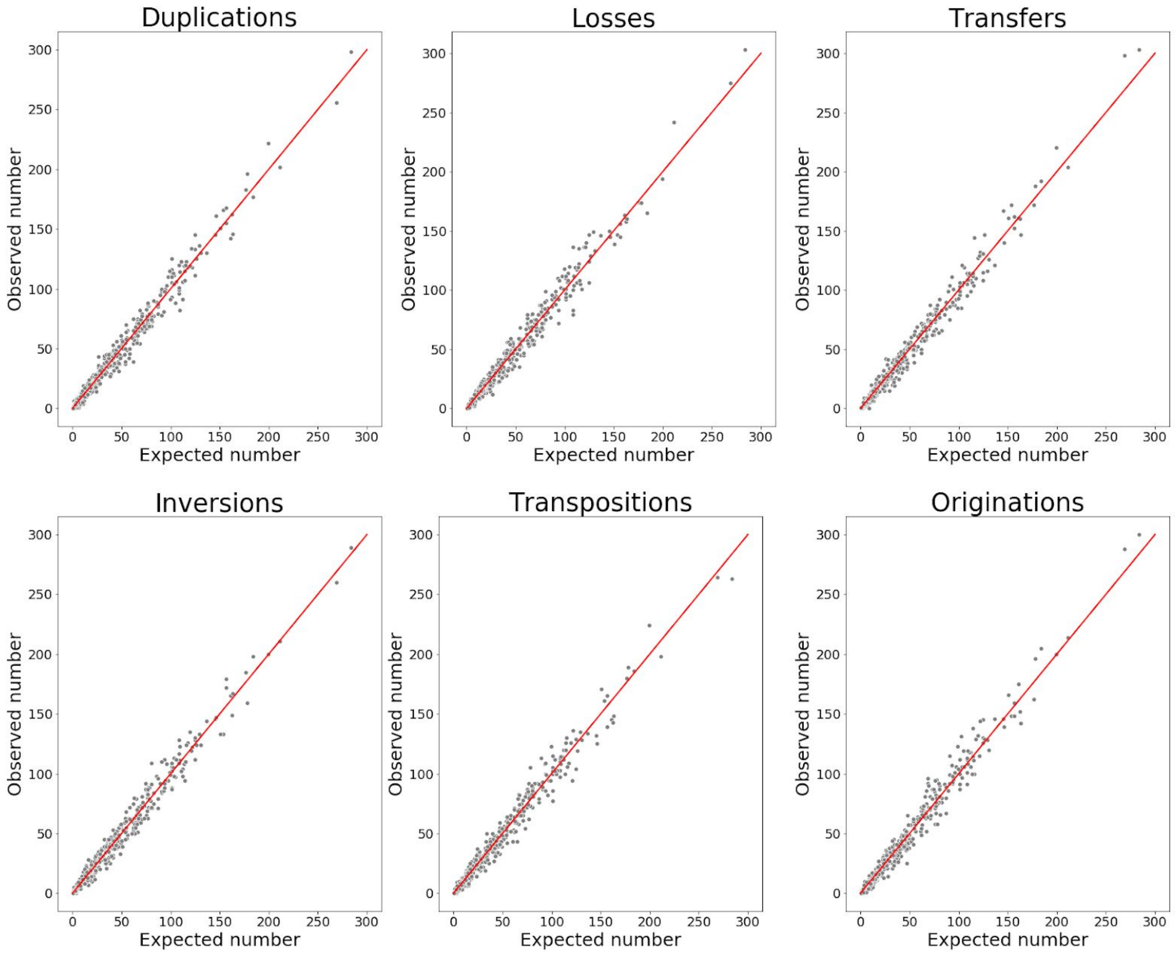

Figure S10: Validation of the rate parameters. We simulate a Species Tree with 100 leaves and the evolution of genomes using the G mode. For every branch of the Species Tree we computed the expected number of events by multiplying the event rate times the branch-length. We plotted the expected number of events against the observed number of events. The red line corresponds to a line with a slope of 1.

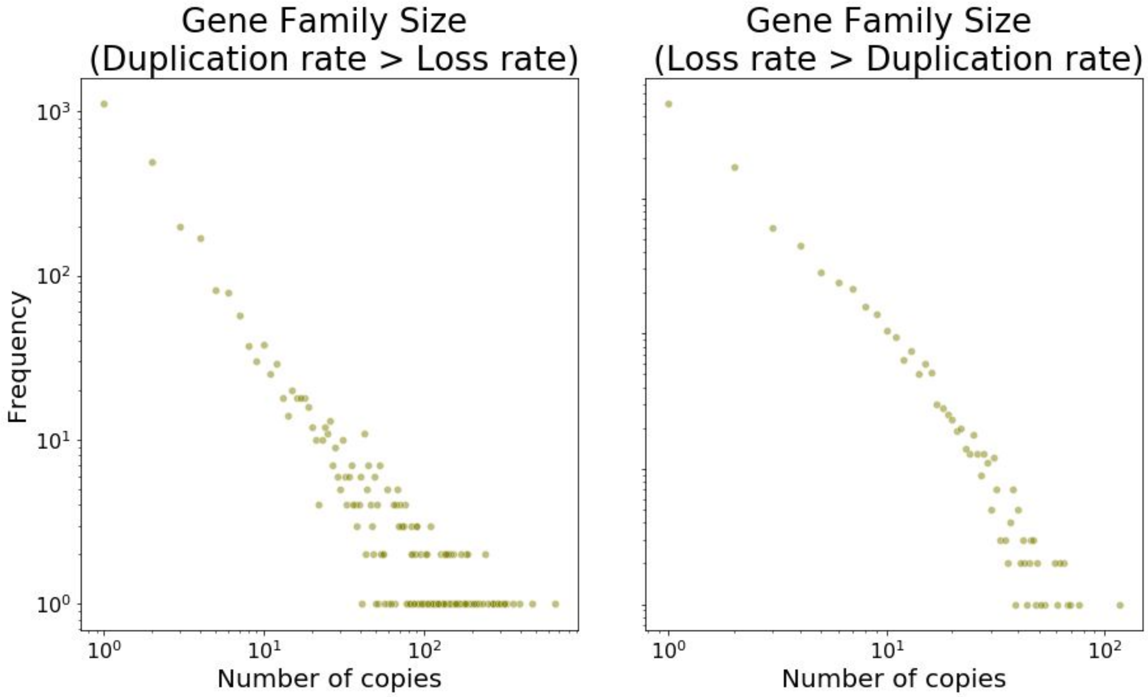

Figure S11: Validation of the mode Gm. In this mode gene families evolve following a birth-death process with specific rates for each family (here we call duplications, D, to births and losses, L, to deaths). It is known that the expected distribution of gene family size follows a power-law distribution when D > L and a stretched exponential if L > D (Szollosi and Daubin 2011; Reed and Hughes 2003). We ran two experiments using the same Species Tree (20 species). In the first one D > L and in the second experiment L > D (the parameter files associated with both experiments can be found in https://github.com/AADavin/ZOMBI/tree/master/Validations. We plotted the frequency against the number of copies for all families with an extant representative in the leaves in log-log axes to inspect visually the distributions and we compared the goodness the support using the Python package Powerlaw (Alstott, Bullmore, and Plenz 2014). We find a clear support for the power-law exponential in the first case (p ~ 3.8^-10) and for the streched-exponential in the second case (p ~5.59 ^ -14)

References

Alstott, Jeff, Ed Bullmore, and Dietmar Plenz. 2014. “Powerlaw: A Python Package for Analysis of Heavy-Tailed Distributions.” *PloS One* 9 (1): e85777.

Beiko, Robert G., and Robert L. Charlebois. 2007. “A Simulation Test Bed for Hypotheses of Genome Evolution.” *Bioinformatics* 23 (7): 825–31.

Dalquen, Daniel A., Maria Anisimova, Gaston H. Gonnet, and Christophe Dessimoz. 2011. “ALF—A Simulation Framework for Genome Evolution.” *Molecular Biology and Evolution* 29 (4): 1115–23.

Kundu, Soumya, and Mukul S. Bansal. 2019. “SaGePhy: An Improved Phylogenetic Simulation Framework for Gene and Subgene Evolution.” *Bioinformatics* , February. https://doi.org/10.1093/bioinformatics/btz081.

Mallo, Diego, Leonardo De Oliveira Martins, and David Posada. 2016. “SimPhy: Phylogenomic Simulation of Gene, Locus, and Species Trees.” *Systematic Biology* 65 (2): 334–44.

Reed, W. J., and B. D. Hughes. 2003. “Power-Law Distribution from Exponential Processes: An Explanation for the Occurrence of Long-Tailed Distributions in Biology and Elsewhere.” *Scientiae Mathematicae Japonicae*. http://citeseerx.ist.psu.edu/viewdoc/download?doi=10.1.1.296.8771&rep=rep1&type=pdf.

Sjöstrand, Joel, Lars Arvestad, Jens Lagergren, and Bengt Sennblad. 2013. “GenPhyloData: Realistic Simulation of Gene Family Evolution.” *BMC Bioinformatics* 14 (June): 209.

Szollosi, Gergely, and Vincent Daubin. 2011. “The Pattern and Process of Gene Family Evolution,” February.
